# The Neuroprotective Effect of *Cornus mas* on Brain Tissue of Wistar Rats

**DOI:** 10.1155/2014/847368

**Published:** 2014-10-16

**Authors:** Renata Francik, Jadwiga Kryczyk, Mirosław Krośniak, Mehmet Berköz, Ilona Sanocka, Sławomir Francik

**Affiliations:** ^1^Department of Bioorganic Chemistry, Jagiellonian University Medical College, 9 Medyczna Street, 30-688 Krakow, Poland; ^2^State Higher Vocational School, Institute of Health, Staszica 1 Street, 33-300 Nowy Sacz, Poland; ^3^Department of Food Chemistry and Nutrition, Jagiellonian University Medical College, 9 Medyczna Street, 30-688 Krakow, Poland; ^4^Department of Pharmaceutical Biotechnology, Faculty of Pharmacy, Yuzuncu Yıl University, Zeve Campus, 65080 Van, Turkey; ^5^Department of Mechanical Engineering and Agrophysics, Faculty of Production Engineering and Energetics, University of Agriculture in Krakow, 116 B Balicka Street, 30-149 Krakow, Poland

## Abstract

Cornelian cherry (*Cornus mas*) is a valuable source of phenolic antioxidants. Flavonoid derivatives as nonenzymatic antioxidants are important in the pathophysiology of many diseases including neurological disorders (e.g., Alzheimer's disease) or heart disease. In this study, we examined the effect of an addition of freeze-dried fruit of cornelian cherry on three types of diets: control diet, fructose diet, and diet enriched in fats (high-fat diet). This effect was studied by determining the following antioxidant parameters in both brain tissue and plasma in rats: catalase, ferric reducing ability of plasma, paraoxonase, protein carbonyl groups, and free thiol groups. Results indicate that both fructose diet and high-fat diet affect the antioxidant capacity of the organism. Furthermore, an addition of cornelian cherry resulted in increased activity of catalase in brain tissue, while in plasma it caused the opposite effect. In turn, with regard to paraoxonase activity in both brain tissue and plasma, it had a stimulating effect. Adding cornelian cherry to the tested diets increased the activity of PON in both tested tissues. Moreover, protective effect of fruits of this plant was observed in the process of oxidation of proteins by decreasing levels of protein carbonyl groups and thiol groups in brain tissue as well as in plasma.

## 1. Introduction

Antioxidant potential of an organism depends on several factors including, for example, type of diet, quantity of consumed vitamins and minerals [1]. Consumption of fruits and vegetables is recommended due to a high content of antioxidants. However, there is a lack of sufficient evidence indicating the necessity of applying special supplementation with antioxidant vitamins [2, 3]. Results of Edwards et al. [4] have shown that balanced energy diets fully cover the demand for vitamins and minerals, and the ratio of pro- and antioxidants in diet remains relatively balanced.

However, a large number of people select products with a high content of sugars, with saturated fatty acids, or with the combination of these components, which is associated with an increased risk of lifestyle diseases. Epidemiologic and experimental data have indicated that changes in the source of lipids consumed in diet may modify fatty acid composition of many cell types [5].

To effectively protect body cells from oxidative stress caused by unbalanced life style and environmental pollution, everyday diet should be enriched with supplements which would be the most efficient and safest source of antioxidants, such as fruits and vegetables containing compounds which decrease the quantity of free radicals generated in the body and especially in the brain.

*Cornus mas* as a product with a high content of antioxidative components, vitamin C, polyphenols, anthocyanins, and minerals [6], can be an interesting supplement to the diet, decreasing systemic oxidative stress.

The high content of vitamin C in its fruits makes it a potential candidate to support treatment and/or prevention of neurological diseases. In fact, the brain is mentioned as one of the organs with the highest concentration of vitamin C. Vitamin C is involved in the process of myelination, and it is also a neuromodulator for neuronal mediators [7]. What is important is that vitamin C is able to penetrate the blood-brain barrier [8]. In course of epilepsy, vitamin C reduces neurodegenerative processes by reduction of lipid peroxidation and, thus, participates in strengthening the cell membrane [9]. Moreover, fruits of* Cornus mas* have numerous health-related properties such as antimicrobial, antiallergic, antihistamine, or antidiabetic ones [1, 10].

In case of well-balanced diet, the consumption of fruits and vegetables, rich in flavonoids, provides the organisms with their sufficient amount *t* (approximately 1 g/day). Nevertheless, most people do not consume a sufficient amount of such food. Therefore, it is necessary to enrich daily diet with supplements containing flavonoids. In addition, diet should also be enriched with elements such as manganese, copper, zinc, iron, and selenium, which facilitate assimilation of flavonoids and enhance their properties.

Resveratrol is a polyphenol present in skin of red grapes. Some results of the research indicated that resveratrol has neuroprotective properties. Among others, it reduces neuronal damage induced by effects of ethanol [11] and protects neurons from toxic effects of beta-amyloid-protein which plays an important role in the development of Alzheimer's disease [12]. In addition, resveratrol significantly reduced kainic acid- (epileptogenous substance-) induced incidence of convulsions [13] as well as death of neurons in hippocampus [14].

Recently, attention has also been paid to green tea neuroprotective properties due to high concentration of flavonoids such a catechin and epicatechin. Epigallocatechin-3-gallate (EGCG) is especially important [15]. Results of behavioral tests in rats (run time, time spent in the probe test) indicate that EGCG inhibits cognitive impairment caused by pentylenetetrazole- (PTZ, epileptogenous substance-) induced epilepsy. That effect may be related to antioxidant properties of EGCG which protects brain cells against free radical damage induced by PTZ. Moreover, this epicatechin effects delay both myoclonic jerks and generalized tonic clonic seizures. The results of the research by Xie et al. demonstrated anticonvulsant properties of EGCG [16].

Another example of a natural substance known as a source of antioxidants is curcumin, a polyphenol from a plant,* Curcuma longa Linn*. Curcumin passes the blood-brain barrier which facilitates its effect on brain neurons [17]. This substance reduces neuronal death by inhibiting caspase-3 and expression of reactive astrocyte. Moreover, curcumin prevents seizures resulting from kainic acid administration [18].

Similarly to curcumin, hydroalcoholic extract of* Emblica officinalis* reduced side effects of administration of pentylenetetrazole, such as seizures, impaired cognitive functions, or oxidative stress [19, 20].

In traditional medicine,* Glycyrrhiza glabra* was used for treatment of epilepsy and its effects are consistent with current results of animal studies [21]. Among bioactive compounds present in the root of* Glycyrrhiza glabra L.,* most of pharmacological properties are attributed to 18-*β*-glycyrrhetinic acid [22]. Ethanol extract from the root of this plant is used in relieving effects of PTZ. The results of the research by Chowdhury et al. demonstrated that polyphenol substances show anticonvulsant potential and ameliorate ROS induced neuronal damage [21]. Neuronal death due to seizures may be the result of excessive production of reactive oxygen species [23]. Nevertheless, increased activity of antioxidant enzymes (catalase, superoxide dismutase) and reduced intensity of lipid peroxidation [21] were observed in brain tissue under the influence of that extract.

Brain tissue is susceptible to oxidative stress due to the high demand for energy, a large amount of lipids and iron as well as catecholamines which are sensitive to oxidation, and lower levels of endogenous antioxidants. In main parts of the brain, the location of catalase is uneven. Only a small part of the whole brain which contains noradrenergic, dopaminergic, and serotonergic neurons is characterized by a particularly high activity of catalase [11].

In this study, the effect of a freeze-dried fruit addition of cornelian cherry to three different diets was evaluated to verify the hypothesis of a protective impact of cornelian cherry on antioxidant status.

## 2. Materials and Method

### 2.1. Animals and Diets

Twelve-week old male Wistar rats, weighting 250 ± 15 g, were used in the experiment. Rats were randomly divided into 6 groups of 6 rats each and acclimatized for 1 week before the main feeding experiment. For 5 weeks, all rats were kept in stainless steel cages with plastic bottom in a room with controlled light for 12 h light/dark cycles, with temperature (23 ± 2°C), humidity (50 ± 10%) kept constant with water ad libitum.

The animals in C− group were given normal rat chow. The composition of each diet is shown in Table 1. Feed in groups C+, F+, and Fa+ was additionally enriched with freeze-dried fruit of* Cornus mas.*

Fruits of this plant came from an experimental orchard of Agricultural University located in Garlica Murowana. From these fruits, stones were mechanically removed and the obtained part pressed to pulp. The processed material was lyophilized in the LIOGAM factory specializing in freeze-drying of fruits and vegetables. The lyophilisate* Cornus mas* powder was added in an amount of 10% by weight of feed which contained all necessary ingredients for proper development of the rats. It has been assumed that the amount of lyophilisate can cover daily demand for raw fruits in humans. These studies were conducted with an approval of I Local Ethics Committee for Animal Experiments of Jagiellonian University number 80/2009 17.09.2009.

### 2.2. Sample Collection and Analysis

At the end of the experiment, after a 16-hour fast, all rats were weighed and euthanized by intraperitoneal injection of sodium thiopental (60 mg/kg) in compliance with requirements of the I Local Ethics Committee. Blood samples were taken from aorta into heparinized tubes and then centrifuged (at 3000 ×g for 15 minutes at 4°C) to obtain plasma which was immediately analyzed or kept frozen (at −80°C) until further analyses. Similarly, brain tissue was rapidly removed, weighed, immediately frozen in liquid nitrogen, and stored at −80°C until further analyses. Then, before the analysis, tissues were homogenized with phosphate buffer (pH 7.4).

### 2.3. Antioxidant Parameters of Rat Brain Tissues and Plasma

The activity of catalase (CAT; EC 1.11.1.6) was determined using the kinetic method by Aebi [24] and estimated in both plasma and brain tissues. The absorbance was read at a wavelength of 240 nm and enzymatic activity was presented as U/g protein. One unit of CAT activity was defined as the amount of enzyme decomposing 1 *μ*mol of H_2_O_2_ per minute.

Paraoxonase enzyme activity (PON; EC 3.1.8.1) was determined by Eckerson's modified method. Paraoxonase activities measurements were performed in the presence of NaCl (salt-stimulated activity). The rate of paraoxon hydrolysis (diethyl-p-nitrophenylphosphate) was measured by monitoring the increase of absorbance at 412 nm at 25°C. The amount of generated p-nitrophenol was calculated from the molar absorptivity coefficient at pH 8.0, which was 18290 M^−1^ cm^−1^. Paraoxonase activity was expressed as U/mg protein in both plasma and brain tissues [25].

The activity of FRAP (ferric reducing ability of plasma) in plasma and brain tissue was measured at 37°C and pH 3.6. Absorbance was measured after 30 minutes and it was proportional to the combined ferric reducing/antioxidant power of the antioxidants in protein. The final results were expressed in mmol Fe^2+^/mg protein [26].

Levels of protein carbonyl groups (PCG) were determined according to the method of Levine [27]. 2,4-Dinitrophenylhydrazine was used as carbonyl group reagent. The absorbance was read at a wavelength of 370 nm. The level of PCG was expressed as nmol/mg protein in both plasma and brain tissue.

Determination of the concentration of free thiol groups (SH) was performed by Hu [28]. The absorbance was read at a wavelength of 412 nm. The level of SH was expressed as mmol/mg protein in both plasma and brain tissue.

### 2.4. Statistical Procedure

Values are given as mean ± standard deviations (SD). Shapiro-Wilks test was applied to check statistical evaluations of biochemical parameters. Statistical differences between the diets (control diet, fructose diet, and high-fat diet) and* Cornus mas* were analyzed by a “two-way ANOVA” test with biochemical parameters difference as the dependent variables and Diet,* Cornus* and Diet with* Cornus*. The critical significance level was set as *P* < 0.05. “Tukey's honestly significant difference” (HSD) test was applied to assess significant differences (*P* < 0.05) between samples. The statistical analysis was conducted using the STATISTICA 10 PL software (StatSoft, Inc.).

## 3. Results and Discussion

Dietary antioxidants are elements which play a particularly important role in decreasing brain damage. Epidemiological data provides information about benefits of the diet rich in antioxidant compounds, which may play an important role in preventing many lifestyle diseases, such as cardiovascular disease, cancer, diabetes, Alzheimer's disease, certain immune disorders, and aging [29, 30]. Anthocyanins are considered as potential scavengers of reactive oxygen species* in vivo* [31]. The plant particularly rich in these molecules is* Cornus mas* [32, 33]. Numerous descriptions of neuro- and cytoprotective activity of anthocyanins, in conditions such as Alzheimer's disease, stroke, and heart attack, can be found in the literature [31].

To find out anti-/prooxidative properties of cornelian cherry in brain tissue and plasma, in our research model standard, fructose and high-fat diet enriched with freeze-dried fruit of cornelian cherry were used (Table 1). What is worth emphasizing is that the effect of an addition of cornelian cherry on oxidative changes, for example, proteins, has not been studied yet. An analysis of this issue may be useful for evaluating the state of cell membrane, particularly in brain tissue.

The basic function of CAT in cells is participation in disproportionation reaction of hydrogen peroxide. Effective removal of excess of peroxide can protect the system from proteins, lipids, and carbohydrates damage.

**Figure 1:**
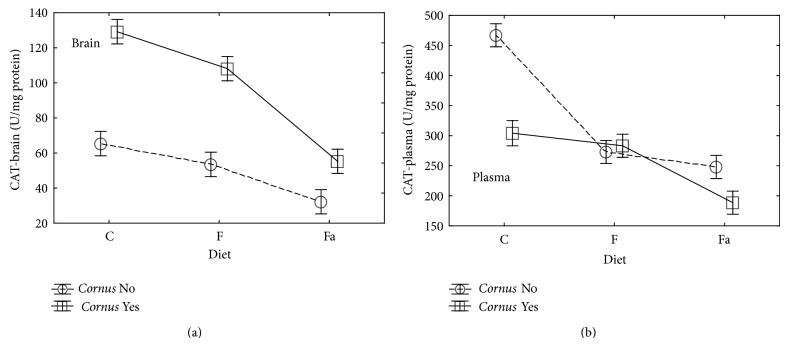


In our experiment, it was observed that an addition of cornelian cherry to control (C+) and high-fat (Fa+) as well as fructose (F+) diet increased the activity of CAT in the brain in a significant way (*P* < 0.05) (Table 2, Figure 1). In the group with a control diet without cornelian cherry (C−), the activity of this enzyme was significant (*P* < 0.05) and higher than in the fructose (F−) and high-fat (Fa−) groups. Combining fructose with cornelian cherry in the feed caused the reduction of the catalase activity in the brain in comparison to the control diet. The same effect was observed in case of the combination of saturated fats with cornelian cherry.

In plasma of the animals fed with the high-fat diet, the activity of CAT decreased statistically (Table 3, Figure 1). Adding fructose to the feed also resulted in the statistically significant decrease of the CAT activity in comparison to the control group. An addition of cornelian cherry to the control and high-fat diets statistically decreased the activity of CAT as compared to that parameter in the C− and Fa− diets.

Comparing results of activity of CAT in brain tissue and plasma, the opposite effect of an addition of cornelian cherry was observed. In brain tissue, activity of this enzyme was increased in the presence of cornelian cherry while in plasma it decreased significantly. It can be assumed that cornelian cherry contains substances which increase protection of the nervous system against oxidative stress.

Catalase activity in the brain may be related to high heterogeneity and location of this enzyme in the central nervous system as well as a high level of its activity related to physiologically important structures, for example, aminergic neurons. Hydrogen peroxide (H_2_O_2_) may be generated in the process of oxidative deamination of biogenic amines under the influence of monoamine oxidase. The ability to produce H_2_O_2_ is also shown by a nitric oxide synthase and ascorbic acid which are present in high concentrations in the brain [34]. However, the yield of synthesis of hydrogen peroxide in these paths is different. Still, another source of H_2_O_2_ can also be a superoxide anion formed with the participation of superoxide dismutase, mitochondrial electron-transporting chain, or cytochrome P450 [35].

Another enzyme the activity of which determines evaluation of oxidative status is paraoxonase-1 (PON1) [36]. It is extracellular esterase linked to high density lipoprotein (HDL) molecules through apolipoprotein A-1 (ApoA1) [37, 38]. PON1 is responsible for anti-inflammatory and antiatherogenic properties of HDL molecules in blood [39]. Low density lipoproteins (LDL), particularly their modified forms, such as oxidized LDL, have proatherogenic properties [40].

**Figure 2:**
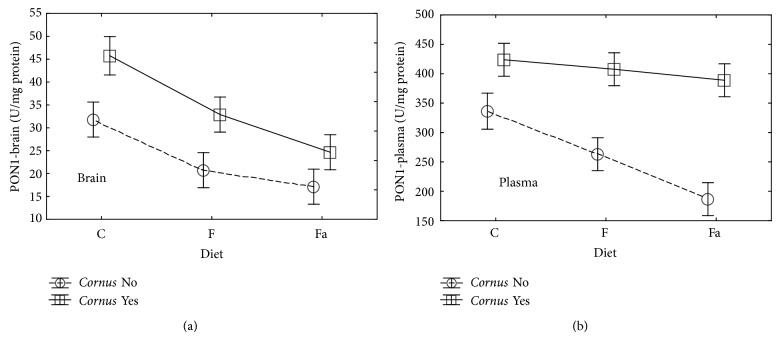


PON is an enzyme from the group of hydrolases, which inhibits oxidation of lipoproteins [39]. PON activity may be beyond genetics, also regulated by environmental factors including diet and availability of antioxidants. Furthermore, it was demonstrated that high-cholesterol-rich diet reduces concentration of PON-1 [41]. Similarly, in our study, a significant (*P* < 0.05) decrease activity of PON in brain tissue and plasma was observed (Tables 2 and 3, Figure 2) in both the fructose (F−) and the high-fat (Fa−) groups compared to the control (C−).

The increase of PON1 activity has a protective effect with regard to the LDL fraction and prevents its oxidation caused by oxidative stress. In addition, Jarvik et al. showed a significant effect of plant components on paraoxonase concentrations in humans [42].

Diet rich in fruits and vegetables increased levels of PON1 in plasma, due to presence of natural antioxidants (vitamins C and E) [42]. Addition of cornelian cherry fruits (“*Cornus* Yes” in the figures) which are rich in polyphenols and vitamin C to each diet also resulted in a significant (*P* < 0.05) increase activity of PON1 both in plasma and in brain tissue. In our research, adding lard to the control feed statistically decreased the value of PON1 both in the brain and in the plasma.

Most enzymes that are involved in oxidation contain iron ions in the structure of heme or iron-sulfur centers. Breach of iron homeostasis and excessive accumulation of those ions in the brain is considered to be a cause of neuronal damage. Changes in oxidation-reduction potential of cells may be the result of chelate metal ions reactions (Cu^2+^, Fe^3+^) [43]. Total antioxidant capability expressed as the ability to reduce the Fe^3+^ to Fe^2+^ was determined by FRAP.

**Figure 3:**
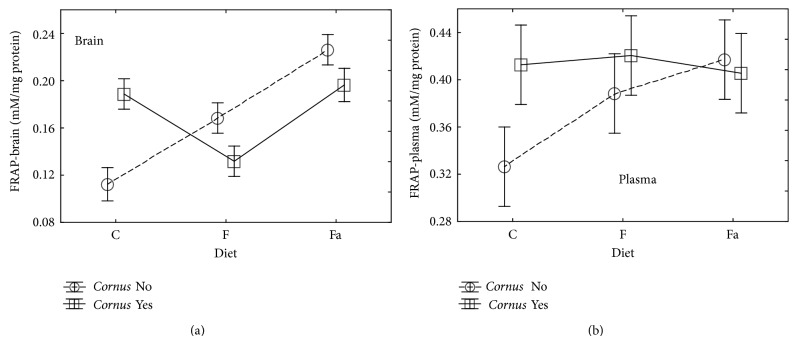


In brain tissue of the animals fed with the fructose or high-fat (lard) diet (Table 2, Figure 3), a statistically significant increase of the FRAP value was observed in comparison to the control group. Adding cornelian cherry to the fructose or high-fat diet caused a statistically significant decrease of the FRAP value in comparison to the groups which did not receive the cornelian cherry addition. The results were different in the control group where the addition of cornelian cherry caused a statistically significant increase of the discussed parameter in comparison to the control diet without this addition. In plasma, the addition of cornelian cherry caused a statistically significant increase of the FRAP value in the control and fructose diet groups.

**Figure 4:**
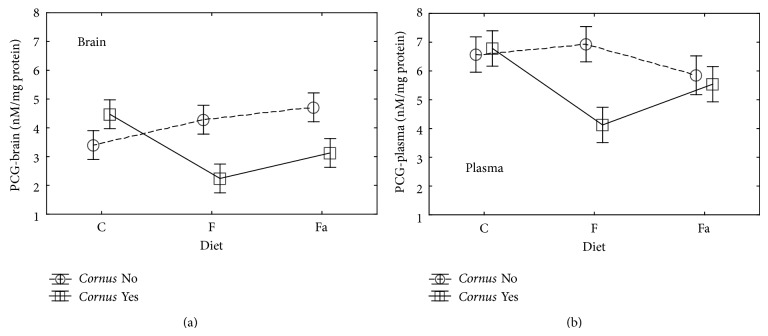


Measurement of carbonyl groups (PCG) was used as a marker of protein damage. Elevated levels of PCG occur in a number of chronic disorders of the central nervous system (CNS), for example, in Alzheimer's disease, Parkinson's in bipolar disorder [44, 45]. One of the proposed mechanisms of increasing the level of PCG is overproduction of a hydroxyl radical which reacts with amino acids, resulting in formation of these groups [46]. In our experiment, a significant (*P* < 0.05) decrease of PCG was observed in the group of rats with F+ in both brain tissue and plasma (Tables 2 and 3, Figure 4). Similar significant dependence (*P* < 0.05) was also observed in brain tissue of rats fed the high-fat feed with an addition of cornelian cherry (Fa+). Polyphenols, anthocyanins, and vitamin C contained in the fruits of cornelian cherry may have a neuroprotective effect on proteins in brain tissue as they reduce the proteins proliferation.

**Figure 5:**
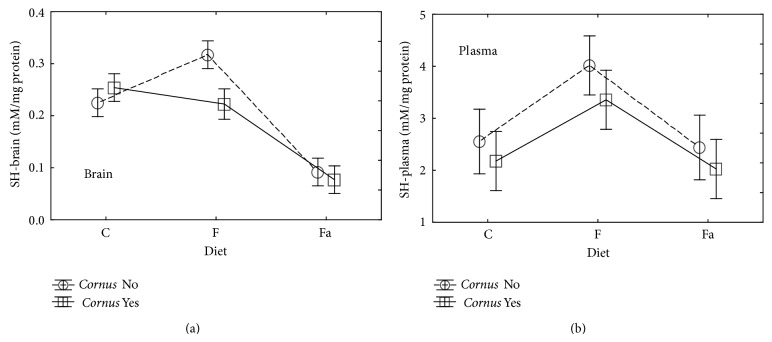


Another marker for evaluation of peroxidation of proteins is concentration of sulfhydryl groups (SH). SH groups are constituents of compounds with antioxidant properties (e.g., glutathione, melatonin, and albumin) undergoing oxidation to disulfide bond (disulfhydryl groups) which reflects the loss of compensatory mechanisms of antioxidant capacity. In this study, in brain tissue (Table 2, Figure 5), a significantly (*P* < 0.05) increased SH groups level was observed in rats fed with fructose diet (F−) compared to the C− and Fa− groups. In turn, in this study, we observed a significant (*P* < 0.05) decrease of this parameter in the Fa− group as compared to the control and fructose diets. Addition of cornelian cherry induced a significantly (*P* < 0.05) decreased SH groups level in rats fed with the fructose diet (F+).

In plasma (Table 3, Figure 5) of rats fed with a fructose diet (F−), a significant increase in levels of SH groups occurred in comparison to the animals of the C− and F− group. Adding cornelian cherry decreases the value of this parameter in all groups but not in a significant way. Based on the obtained results, it can be assumed that a content of SH groups in plasma is affected by the type of diet but not by an addition of cornelian cherry.

Oxidative modifications of proteins are the fastest emerging indicator of oxidative damage in cells, demonstrating disorder of redox balance. This is due to the fact that proteins are not only chemical reactants, but also catalysts for many processes in body. Therefore, changes in their structure and function modifications are much larger than in case of other biomolecules. All this confirms the validity of the selection of oxidative damage markers for proteins (carbonyl groups, SH groups).

## 4. Conclusion

The disruption of antioxidant balance of the body system is an important factor in development of many diseases, including neurologic ones, due to the fact that brain tissue is very sensitive to oxidative stress. Cornelian cherry contains many substances with antioxidant properties. Moreover, based on the results of our study, it can be assumed that an addition of cornelian cherry advantageously stimulates PON1 activity both in brain tissue and in plasma and increases protection of the nervous system from oxidative stress by increasing activity of CAT. At the same time, it protects proteins against peroxidation as can be shown by the level of PCG. Thus, the above results indicate that cornelian cherry may be a natural source of neuroprotection. However, it is necessary to continue this research.

## Figures and Tables

**Table 1:** The composition of experimental diets.

Components	Control diet (C) %	Fructose diet (F) %	Fatty diet (Fa) %
Starch	62	32	32
Casein	20	20	20
Oil	5.0	5.0	5.0
Lard	0	0	30
Fructose	0	30	0
Calcium carbonate	2.8	2.8	2.8
Ca_3_(PO_4_)_2_	2.9	2.9	2.9
Lecithin	1.0	1.0	1.0
NaCl	0.3	0.3	0.3
Cellulose	4.7	4.7	4.7
Minerals and vitamins mix.	1.0	1.0	1.0
MgO	0.07	0.07	0.07
K_2_SO_4_	0.23	0.23	0.23

**Table 2:** Activity of oxidative stress markers (CAT, PON1, FRAP, PCG, and SH) marked in brain tissue homogenates in Wistar rats. Data are presented as means from independent measurements ± standard deviation (SD). Different letters in the same columns indicate significant differences according to Tukey's test (*P* < 0.05).

Diet	*Cornus*	CAT-brain U/mg protein	PON1-brain U/mg protein	FRAP-brain mM Fe^2+^/mg protein	PCG-brain nM/mg protein	SH-brain nM/mg protein
C−	No	65.4 ± 17.9^B^	31.8 ± 14.3^AB^	0.112 ± 0.015^A^	3.4 ± 1.47^AB^	0.225 ± 0.091^A^
F−	No	53.5 ± 20.6^AB^	20.7 ± 6.5^A^	0.168 ± 0.044^AB^	4.28 ± 1.37^AB^	0.317 ± 0.089^A^
Fa−	No	32.2 ± 13.7^A^	17.1 ± 7.2^A^	0.226 ± 0.017^C^	4.71 ± 1.51^A^	0.092 ± 0.013^B^
C+	Yes	129.2 ± 21.2^C^	45.8 ± 8.4^B^	0.189 ± 0.041^BC^	4.47 ± 1.23^A^	0.254 ± 0.084^A^
F+	Yes	108.1 ± 8.5^C^	32.9 ± 11.6^AB^	0.132 ± 0.018^A^	2.24 ± 0.82^B^	0.222 ± 0.035^A^
Fa+	Yes	55.3 ± 16.6^AB^	24.7 ± 4.4^A^	0.196 ± 0.038^BC^	3.13 ± 0.73^AB^	0.077 ± 0.021^B^

C−: control; F−: fructose; Fa−: high fat; C+: *Cornus* with control; F+: *Cornus* with fructose; (Fa+): *Cornus* with high fat.

**Table 3:** Activity of oxidative stress markers (CAT, PON1, FRAP, PCG, and SH) marked in plasma in Wistar rats. Data are presented as means from independent measurements ± standard deviation (SD). Different letters in the same columns indicate significant differences according to Tukey's test (*P* < 0.05).

Diet	*Cornus*	CAT-plasma U/mg protein	PON1-plasma U/mg protein	FRAP-plasma mM Fe^2+^/mg protein	PCG-plasma nM/mg protein	SH-plasma nM/mg protein
C−	No	467.1 ± 56^C^	336.3 ± 71.6^AC^	0.326 ± 0.065^A^	6.57 ± 1.94^AB^	2.554 ± 0.265^A^
F−	No	272.9 ± 21.7^A^	263.1 ± 73^BC^	0.388 ± 0.075^A^	6.93 ± 1.96^A^	4.017 ± 1.226^A^
Fa−	No	248 ± 23.4^AB^	186.6 ± 23.1^B^	0.417 ± 0.146^A^	5.85 ± 0.64^AB^	2.44 ± 0.642^A^
C+	Yes	304.1 ± 69.4^A^	423.7 ± 98.8^A^	0.413 ± 0.028^A^	6.78 ± 0.85^A^	2.179 ± 0.968^A^
F+	Yes	283.2 ± 41.8^A^	407.6 ± 64.7^A^	0.421 ± 0.085^A^	4.12 ± 1.01^B^	3.356 ± 2.697^A^
Fa+	Yes	188.6 ± 55.4^B^	388.8 ± 58.1^A^	0.406 ± 0.042^A^	5.54 ± 1.85^AB^	2.026 ± 0.85^A^

C−: control; F−: fructose; Fa−: high fat; C+: *Cornus* with control; F+: *Cornus* with fructose; Fa+: *Cornus* with high fat.

## Abbreviations

ApoA1: Apolipoprotein A-1
CAT: Catalase
EGCG: Epigallocatechin-3-gallate
FRAP: Ferric reducing ability of plasma
HDL: High density lipoprotein
LDL: Low density lipoprotein
PCG: Protein carbonyl groups
PON: Paraoxonase enzyme
PTZ: Epileptogenous substance
SH: Thiol groups.
